# The Mental Representation of Polysemy across Word Classes

**DOI:** 10.3389/fpsyg.2018.00192

**Published:** 2018-02-21

**Authors:** Anastasiya Lopukhina, Anna Laurinavichyute, Konstantin Lopukhin, Olga Dragoy

**Affiliations:** ^1^Neurolinguistics Laboratory, National Research University Higher School of Economics, Moscow, Russia; ^2^Vinogradov Institute of Russian Language, Russian Academy of Sciences, Moscow, Russia; ^3^Department of Linguistics, University of Potsdam, Potsdam, Germany; ^4^Scrapinghub, Moscow, Russia; ^5^Department of Speech Pathology and Neurorehabilitation, Moscow Research Institute of Psychiatry, Moscow, Russia

**Keywords:** polysemy, lexical representation, metaphor, metonymy, semantic vectors, word classes

## Abstract

Experimental studies on polysemy have come to contradictory conclusions on whether words with multiple senses are stored as separate or shared mental representations. The present study examined the semantic relatedness and semantic similarity of literal and non-literal (metonymic and metaphorical) senses of three word classes: nouns, verbs, and adjectives. Two methods were used: a psycholinguistic experiment and a distributional analysis of corpus data. In the experiment, participants were presented with 6–12 short phrases containing a polysemous word in literal, metonymic, or metaphorical senses and were asked to classify them so that phrases with the same perceived sense were grouped together. To investigate the impact of professional background on their decisions, participants were controlled for linguistic vs. non-linguistic education. For nouns and verbs, all participants preferred to group together phrases with literal and metonymic senses, but not any other pairs of senses. For adjectives, two pairs of senses were often grouped together: literal with metonymic, and metonymic with metaphorical. Participants with a linguistic background were more accurate than participants with non-linguistic backgrounds, although both groups shared principal patterns of sense classification. For the distributional analysis of corpus data, we used a semantic vector approach to quantify the similarity of phrases with literal, metonymic, and metaphorical senses in the corpora. We found that phrases with literal and metonymic senses had the highest degree of similarity for the three word classes, and that metonymic and metaphorical senses of adjectives had the highest degree of similarity among all word classes. These findings are in line with the experimental results. Overall, the results suggest that the mental representation of a polysemous word depends on its word class. In nouns and verbs, literal and metonymic senses are stored together, while metaphorical senses are stored separately; in adjectives, metonymic senses significantly overlap with both literal and metaphorical senses.

## Introduction

Polysemy is one of the fundamental properties of the lexical system of a language. The most common words of a language are polysemous; that is, they have a number of related senses^1^ (Zipf, 1945). Psycholinguistic research of polysemy addresses two major questions: how senses of a word are stored in the mental lexicon and how they are processed during language comprehension. Available studies provide contradictory evidence. While some studies argue in favor of separate sense storage (Klein and Murphy, 2001, 2002; Foraker and Murphy, 2012), others present evidence of sense overlap and underspecified core representations in the mental lexicon^2^ (Frazier and Rayner, 1990; Frisson and Pickering, 1999; Pickering and Frisson, 2001). The current study uses a semantic clustering approach to address the issue of whether different senses of polysemous words (Russian nouns, verbs, and adjectives) are stored as separate or shared mental representations.

### Theoretical Studies on Polysemy

Polysemy is not a uniform phenomenon. Theoretical linguists traditionally divide the multitude of a word’s senses into literal, metonymic, and metaphorical senses (Apresjan, 1974; Pustejovsky, 1995; Geeraerts, 2010). Metonymic shifts occur within the semantic domain of the literal sense and are driven by contiguity between the senses. For example, while talking about a *crocodile* handbag, one focuses on the leather produced from the animal skin rather than on the animal as a whole, but stays within the same animal domain (Lakoff and Johnson, 1980; Peirsman and Geeraerts, 2006; Littlemore, 2015). Metonymically motivated polysemy is not accidental and follows a large number of typical patterns, such as animal/food (*tasty rabbit*), container/containee (*drink a bottle*), producer/product (*read Shakespeare*), or place/people (*Germany signed a treaty*). Many types of metonymic shifts seem to hold cross-linguistically: the above mentioned English patterns are also consistent for Russian (see Apresjan, 1995). The systematic nature of metonymic shifts makes metonymy predictable and productive. Speakers can use familiar patterns to create novel senses that they have never encountered before (Murphy, 1997), such as *I read Alice Munro*. Nunberg (1979) argues that metonymy is a natural referential strategy used when one cannot point at the referent itself, but can identify it by pointing at something else that stands in a certain relation to the referent. Metonymic senses are interpretable and perceived as related to the literal senses from which they are derived.

Polysemy may also be motivated by metaphor. Metaphor is the mechanism for seeing one thing in terms of another. A new sense is derived from the literal sense of a word through metaphorical mapping: the word’s existing sense is transferred from its own source domain to another target domain, based on structural similarities between the domains (e.g., if one calls an aggressive opponent a *crocodile*, s/he metaphorically maps the animal domain onto the human domain). Usually, concrete and embodied word senses are extended to more abstract senses (Lakoff and Johnson, 1980; Johnson, 1987; Lakoff, 1987; Geeraerts, 2010; Xu et al., 2017). For example, the word *to catch* can be used in the original literal sense of ‘to stop and hold something that is moving through the air’ in collocations like *to catch a ball, to catch a butterfly*. However, the idea of stopping and getting something can be extended to express an abstract metaphorical sense of falling ill, such as in the collocations *to catch the flu, to catch chickenpox*. Metaphorical polysemy is based on similarity, but the relations between literal and metaphorical senses are not always obvious to speakers (Apresjan, 1974) and can be confused with homonymy.^3^ For example, some Russian dictionaries treat the two meanings of the word *duma* ‘thought’/‘parliament’ as homonyms (Kolesnikov, 1978; Shvedova, 2007), while others see them as polysemic (Akhmanova, 1976; Kuznetsov, 1998).

However, some researchers argue that the dividing line between metonymy and metaphor is not clear-cut: the relation between them is still debated, especially within the cognitive linguistics framework (Barcelona, 2000; Benczes et al., 2011; Littlemore, 2015). For example, Radden (Barcelona, 2000) claimed that metonymy and metaphor are two endpoints on a continuum with unclear cases in between. The fuzzy middle range of the continuum can be treated as metonymy-based metaphors — mappings that involve two conceptual domains grounded in one conceptual domain (like *high prices*). The idea of a continuum is also mentioned by Dirven (2003), who considered metonymy and metaphor as sitting on a continuum ranging from literal language to metaphor, with metonymy in the middle. Moreover, Barcelona (Barcelona, 2000) argues that every metaphorical mapping presupposes a prior conceptual metonymic mapping, such that all metaphors are grounded in metonymies. Metonymy and metaphor often interact with each other so that sometimes it is not easy to say with certainty whether an observed non-literal sense of a word is to be regarded as metonymic or metaphorical. Overall, there is still no agreement on how to distinguish metonymy from metaphor or whether it should be done at all. In this study, we consider metonymy as mapping within the same conceptual domain, and metaphor as mapping between conceptual domains, and work with clear-cut cases where metonymy and metaphor are distinguishable.

Having identified the relations that hold among multiple senses of polysemous words, the question arises as to how these senses are mentally represented. Theoretical linguists have long debated about the problem of sense representation in the mental lexicon and proposed several competing theories (for an overview, see Zaliznyak, 2006; Geeraerts, 2010). The basic opposition is that of separate sense representations vs. a single core representation. In the separate sense account, words are assumed to have an exhaustive list of senses that are stored in the mental lexicon and accessed during language processing (Weinreich, 1966; Kempson, 1977; Clark and Gerrig, 1983). This type of representation implies that a person immediately selects one intended sense when processing a polysemous word. However, the separate sense account is problematic when it comes to sense storage and processing novel or occasional senses. First, separately storing each sense of a word is not economical; according to this account, the verb *to see* should have 45 representations (LDOCE^4^). And second, speakers are able to create novel extensions of words in context that can be understood without being pre-stored (e.g., *John left after Pokémon Go*, where the name of the game is used in the ‘event’ sense); this contradicts the separate sense account. In addition, the separate sense account introduces a complication of distinguishing word senses that has been widely discussed in lexicographic and computational linguistics literature (see Kilgarriff, 1997; Wilks, 1998; McCarthy et al., 2016).

The alternative single sense account assumes that there is one core representation of each word in the mental lexicon (Caramazza and Grober, 1976; Nunberg, 1979). Specific senses of a word are constructed based on context and patterns of extension, such as the animal/food metonymic pattern for the words *chicken, lamb, fish*, etc. A similar idea was proposed by Pustejovsky (1995) as a “generative lexicon” (see also Jezek, 2016). According to Pustejovsky, the lexicon is a generative system: context-relevant word senses are derived from the core representation with the help of lexical rules. Both the core meaning and the derivation rules are stored in the mental lexicon. Storing a limited amount of lexical semantic information is economical, though it requires more time and processing power to derive the particular sense intended in an utterance. However, according to Klein and Murphy (2001), closely related senses may not be conceptually similar to each other. For example, the word *church* can refer to a building (*The church burned down*) or to an organization (*The church has lost many members*) and shares the building/organization metonymic pattern with words like *school, bank*, and *hospital*. Although the ‘building’ and ‘organization’ senses are closely related, the two concepts do not have much in common: buildings are built of physical materials, are of a certain height, and have a color and weight, while organizations unite people who have similar beliefs. Therefore, semantic relatedness does not necessarily lead to semantic similarity, and this conceptual incoherence is problematic for the single sense storage account (see also Foraker and Murphy, 2012).

A hybrid approach to sense storage is possible. Polysemy is a broad phenomenon and thus can embrace different cases: some senses are closer than others. As Deane (1988) noticed, some senses (based on metonymies) can be created from the core representation when meaning is tailored to context, while others (based on metaphor) may have not much in common with literal senses. Therefore, different senses of a polysemous word might not be uniformly stored either as a single core or separate representations. The frequency of a sense may also be an important factor: frequent semantic extensions are easier to keep in separate representations (see Kawamoto, 1993; Klein and Murphy, 2002). Tuggy (1993) proposed a model in which the difference between word senses is gradual and a polysemous word may have both the core representation and several separate senses in the mental lexicon. Similarly, Zaliznyak (2006) argued that different words may have different representations: words with few senses may be stored as a single core representation, while highly polysemous words can have several representations in the mental lexicon.

### Experimental Studies on Polysemy

In the psycholinguistic literature, the question of polysemy representation has received a great deal of attention. Some experimental studies have provided evidence for separate sense storage. The first piece of evidence in favor of the separate storage account was presented by Klein and Murphy (2001). Participants were asked to judge as quickly as possible whether phrases made sense. The phrases were presented in pairs one after the other and contained the same polysemous word either in the same or in a different sense: *wrapping paper/shredded paper* — the same sense; *shredded paper*/*daily paper* — different senses. The results showed that in the same sense condition participants were more likely to judge the phrase as making sense, and they tended to make this decision faster than in the different senses condition: processing the word in one sense did not enhance processing it in another sense. This finding could be explained by an active process of inhibition: in the different senses condition, the sense of the first phrase must be suppressed to activate the appropriate sense of the second phrase. Klein and Murphy concluded that their results are in line with the separate storage account and that senses of a word should have minimal semantic overlap (see also the behavioral results of Pylkkänen et al., 2006). The research was extended by Klein and Murphy (2002) in a study that examined the closeness of word senses in a categorization task. The results were consistent with the previous findings: participants preferred not to group together phrases with a polysemous word in different senses. According to the authors, it means that senses of a word are represented separately, with little semantic overlap. One more piece of evidence for the idea of separate sense storage was presented by Foraker and Murphy (2012). They performed a study that involved eye tracking while reading, in which context and sense frequency were manipulated: *The fashion designers discussed the cotton… (fabric)/The farm owners discussed the cotton… (crop)/They discussed the cotton… (fabric or crop)*, where the beginning of the first sentence disambiguates for the dominant sense, the beginning of the second disambiguates for the subordinate, and the beginning of the third (neutral) phrase remains ambiguous. In all of the conditions a polysemous word was followed by a disambiguating phrase. It was found that in the neutral condition, the disambiguating phrase was read faster when it referred to the dominant and not the subordinate sense. The results are most consistent with the claim that the dominant sense, and not the underspecified core meaning equally compatible with all senses, is accessed. The authors concluded that readers select an individual sense when reading a polysemous word, and this is consistent with the separate sense account (but see Frisson, 2015 for a discussion).

In contrast with the above mentioned papers, several experimental studies provide evidence for the single sense account. Most of the studies involved eye tracking while reading. Based on participants’ reading times of the two senses of polysemous words, Frazier and Rayner (1990), Frisson and Pickering (1999), Pickering and Frisson (2001), Frisson and Frazier (2005), and Frisson (2009, 2015) have argued that in ambiguous contexts readers initially activate a single semantically underspecified meaning of a word that encompasses all related interpretations known to the reader. The claim was based on the absence of extra processing costs for the less frequent senses in ambiguous contexts. Frisson and Pickering (1999) suggested that an underspecified meaning becomes specific later, in a subsequent commitment (‘homing-in’) stage. Furthermore, the behavioral experiments of Rodd et al. (2002) showed that highly polysemous words were recognized faster than polysemous words with few senses or homonyms in a lexical decision task. The authors concluded that the rich semantic representations associated with highly polysemous words facilitated their recognition, and that unlike homonyms, senses of a polysemous word do not compete for the activation of their individual semantic representations, but instead speed up the processing of one another. These results are in line with the single sense account. Later experiments showed that the observed processing advantage can be modulated by sense relatedness. For example, Brown (2008) demonstrated a correlation between sense relatedness and processing speed: short phrases with closely related senses were judged as making sense with almost the same speed and accuracy as phrases with the same sense. This suggests that closely related senses may be represented in the mental lexicon similarly to a single sense, perhaps sharing a core semantic representation. Brown noticed that closely related senses distinguish between different concrete usages of a word, whereas the distantly related senses distinguish between a concrete and a figurative or metaphorical usage. Therefore, both the degree and the type of relatedness are probably correlated and may affect polysemy processing.

The hybrid approach to sense storage and differences in processing various types of polysemy was largely supported by experiments that directly compared metaphorical and metonymic senses. For example, Klepousniotou (2002) found that in a priming lexical decision task metonymically polysemous words demonstrated a stronger facilitation effect and were recognized as existing words faster than metaphors. These results were supported by the study of Klepousniotou and Baum (2007) who found that the processing of polysemous words depends on the type of sense relations: metonymies elicited faster reaction times than metaphors. Klepousniotou et al. (2008) used the paradigm of Klein and Murphy (2001) with cooperating/conflicting/neutral contexts to study the effects of sense overlap and sense dominance on polysemy processing. They found that in conflicting contexts words with highly overlapping senses (metonymy) were judged as meaningful faster and more accurately than words with moderately overlapping senses (metaphor). Moreover, Klepousniotou et al. (2012) and MacGregor et al. (2015) found an effect of the type of polysemy on the time-course of sense activation in electrophysiological experiments. All of these studies support the division of polysemy into metaphor and metonymy, as recognized in theoretical linguistics, and suggest that metonymies are stored in the same mental representations as literal senses, while metaphors are stored as separate representations. However, Jager and Cleland (2015) reported the opposite pattern: metaphors were recognized faster than metonymies, suggesting that metaphorical and metonymic senses can be stored similarly in the mental lexicon.

The heterogeneity of the available evidence on the representation and processing of polysemous word senses can be attributed to specific tasks and materials used in the studies. Task demands change the way we extract information from a stimulus. For example, Chen et al. (2013) showed that task-specific processes started to penetrate word recognition as early as at 150 ms after the stimulus onset, thus inevitably affecting the target outcome behavioral measure. Previous experimental studies of polysemy mostly used online methods: either priming lexical decision or eye tracking while reading. These tasks measure different kinds of processing: lexical decision focuses attention on the sensicality of an item, while sentence reading involves pragmatics and more closely resembles natural language processing. Mahé et al. (2015) showed that lexical decision and reading tasks differ in their core processes as they involve different amounts of neural resources. However, it is possible that neither task involves deep semantic processing: in lexical decision, participants are instructed to react as fast as they can and thus they might not have enough time to process the semantics of stimuli; in reading, they do not focus on a single word in question but target the integral meaning of an utterance/discourse. In contrast, an offline categorization task proposed by Klein and Murphy (2002) did not force participants to answer quickly and allowed them to discover even subtle differences in sense relatedness. This enabled the researchers to detect small semantic overlaps between the senses of polysemous words, while the lexical decision task used by Klein and Murphy (2001) was not sensitive enough to capture this overlap. Although offline methods measure only the result of semantic processing, they simultaneously allow participants to spend as much time as they need deciding about each stimulus. Hence, if participants do not label two senses as different when they could process the senses for as long as they chose, we can conclude that participants genuinely do not distinguish between these senses. And it is likely that senses that are not distinguished under the most favorable circumstances are stored together.

Previous studies also had a methodological limitation: during the experiment, each word was usually presented in one sentence or two short phrases. Such limited contexts may not be prototypical representatives of the literal/metonymic/metaphorical senses, which can influence participants’ responses. Context is known to strongly influence perceived meaning (Hanks, 2000; McDonald and Ramscar, 2001), and contextual similarity underlies the research area of distributional semantics in which semantic similarities between linguistic items are discovered based on their distributional properties in large corpora (Firth, 1957; Schütze, 1993). Therefore, potentially shallow semantic processing and limited contexts might have distorted previously obtained results. The method we used in the current study aims to overcome such limitations.

### The Present Study

The present study aims to investigate the closeness of literal, metonymic, and metaphorical senses, in order to inform theories of polysemous word representation in the mental lexicon. It extends the above mentioned studies in a number of different directions. First, we used a new experimental paradigm: to find out which of the three types of senses are perceived as related, we asked participants to cluster experimental stimuli together into virtual baskets. This approach implies the involvement of deep semantic processing, similar to the categorization task exploited by Klein and Murphy (2002) and Jager and Cleland (2015), but critically differs from those studies in terms of available choice alternatives and contextual limitations. The previous studies used forced-choice sorting tasks. In Klein and Murphy (2002), participants were given a target phrase that included a polysemous word in one sense (*wrapping paper*) and two other phrases for matching to the target: one with the same polysemous word but used in a different sense (*liberal paper*), and another with a new word taxonomically or thematically related to the target (*smooth cloth*). In Jager and Cleland (2015), participants were shown a word (e.g., *lion*) and asked to decide whether it was an inanimate object or an animal. Thus, the number of alternatives was artificially restricted to two. However, participants may not prefer any of the presented alternatives, and since they are forced to choose something, they agree to the lesser of two evils instead of making their own categorization. We developed a paradigm that allows participants to sort stimuli according to their individual representations by creating an unlimited number of categorization groups. Moreover, in the proposed paradigm participants saw multiple contexts of a polysemous word (at least 6 for each word). This solved the problem of restricted context, which was typical in previously used paradigms.

The second extension of the current study concerns a direct comparison of sense relatedness and sense similarity measures of the literal, metonymic, and metaphorical senses of polysemous words. Klein and Murphy (2001) argued that sense relatedness did not necessarily lead to conceptual similarity; the two senses may be derived one from the other and thus be closely related but at the same time they may be dissimilar (e.g., *wrapping paper* that refers to a physical object is not similar to *boring paper* that refers to content). We aimed to distinguish between or link the concept of sense relatedness that reflects the historic development of the polysemy, on one hand, and that of sense similarity that shows how much the two senses overlap, on the other hand. While sense relatedness measures were to be obtained from the experiment, in order to quantify sense similarity we exploited the corpora-based methodology of distributional semantics (Firth, 1957; Lenci, 2008), conceptualizing semantic similarity as an ‘is a’ relation and reflected in the probability of two lexical items (words or multiword expressions) to be interchangeable in the same context (for a comprehensive overview of vector semantics, see Jurafsky and Martin, 2017). Measures of semantic similarity in our study were extracted from text corpora using semantic vector analysis (Turney and Pantel, 2010; Mikolov et al., 2013a,b), widely used in the natural language processing domain in such areas as the identification of lexical semantic relations (synonymy, antonymy, hypernymy, part-whole meronymy), text similarity estimation, word sense disambiguation, and question answering. For the first time, we estimated the contextual distribution of the literal, metonymic, and metaphorical senses of polysemous words in order to quantify the similarity of their semantic content. Then we compared the extracted measures of different senses’ distributional similarity with the results of the categorization experiment, thus directly contrasting sense similarity and sense relatedness.

Another novelty of the present experiment is the stimulus materials. The majority of previous studies focused on nouns; some, like Klein and Murphy (2001) and Rodd et al. (2002), used both nouns and verbs. However, in two eye tracking reading experiments with verbs, Pickering and Frisson (2001) found that the processing of verbs differed from that of nouns. In addition, there is ample evidence that verbs and nouns are represented in segregated neural networks (Vigliocco et al., 2011) and that verbs are harder to process. Verbs have lower recall rates than nouns in memory tasks (Szekely et al., 2005), are acquired later (Bassano, 2000), are harder to learn (Childers and Tomasello, 2006; Gentner, 2006). It is probable that verbs also differ from nouns in the way their senses are stored, which requires more focused investigation. In addition, experiments with polysemy have never used adjectives as stimuli — they only served to disambiguate target nouns. It appears that nothing is known about the processing of polysemous adjectives. In the current study, we used nouns, verbs, and adjectives as stimuli and analyzed the three word classes separately in order to test whether the conclusions inferred from noun studies can be generalized to other word classes.

Yet another extension of our study is a more careful approach to the selection of participants. We found it to be important to separate participants with linguistic background from those with other backgrounds. Previous experiments did not distinguish between linguists and non-linguists; however, the participants’ background may influence their decisions about word senses. Professional linguists are trained to think about polysemy in terms of metonymy and metaphor, and may sort senses according to their formal knowledge. Non-linguists’ classifications instead might be guided by other (e.g., usage-based) principles. To study the possible difference between speakers with and without linguistic backgrounds, we analyzed the responses of professional linguists and non-linguists (naive language speakers) separately. We expected that professional linguists would classify different senses of polysemous words into literal/metonymic/metaphorical senses like professional lexicographers do, while the classification of naive participants might diverge from that pattern. Although all naive participants have basic ideas about metonymy and metaphor, as taught in Russian schools, we did not expect that knowledge to be actively engaged during classification.

To conclude, the current study investigates the sense relatedness and sense similarity of Russian nouns, verbs, and adjectives in two groups of participants: linguists and non-linguists. In contrast to previous studies, we used an unrestricted sorting task and an extended set of stimuli.

## Materials and Methods

The study exploited two methodological approaches: a semantic clustering experiment and corpora-based semantic vector analysis. In this section, the experimental method and two experimental analyses are described (see section “Semantic Clustering Experiment”), and a vector analysis of the experimental stimuli is presented (see section “Semantic Vector Analysis”).

### Semantic Clustering Experiment

#### Participants

2,080 volunteers, self-reported native speakers of Russian with no neurological/psychiatric disorders, were recruited through mailing lists and social networks to participate in an online experiment. 840 participants completed the entire experiment; 1,240 participants completed it partly, and their data were also included in the analysis. Before proceeding to the experiment, participants were asked to provide information on their native language(s), sex, age, dominant hand, profession and the last obtained degree. 8 of the 2,080 participants were male^5^; the age of participants ranged between 17 and 70 years, with a mean of 27 (*SD* = 9); 1,842 participants were right-handed, 103 were left-handed, and 143 participants were ambidextrous. Based on the reported professions and the last obtained degrees, participants were categorized either as professional linguists or as naive individuals (with respect to linguistic knowledge). All participants who had at least a bachelor’s degree in linguistics, philology, journalism, or translation studies were considered to be professionals (*N* = 727), while all other individuals were considered to be naive.^6^

#### Materials

Thirteen professional lexicographers [the group of Jury Apresjan that works on the *Active Dictionary of Russian* (Apresjan, 2014)] selected 48 Russian polysemous words (24 nouns, 12 verbs, and 12 adjectives), each with literal, metonymic and metaphorical senses; the lexicographers described the senses and designed short phrases representing each of the senses (at least two phrases for each sense of a word). The words were selected as follows: each lexicographer suggested 5–10 polysemous words with literal, metonymic and metaphorical senses; then everyone independently rated all 92 words. Words that obtained 100% agreement were used in the experiment. All short phrases with target words were designed by lexicographers based on examples from the Russian National Corpus^7^. Overall, each polysemous word was introduced in 6–12 short phrases that represented three to six separate word senses. Only 30 words had three senses (literal, metonymic, and metaphorical; see sample senses and their representative phrases for the three word classes in **Table 1**), while the rest of the words had either two literal or two metonymic or two metaphorical senses. Four words had five senses (the nouns *knizhka* ‘book’ and *scena* ‘stage,’ the verb *zavalit’* ‘to cover,’ and the adjective *kislyj* ‘sour’) and the nouns *okno* ‘window’ was presented in six senses: one literal, two metonymic and three metaphorical.

**Table 1:** Three examples of the stimuli for nouns, verbs, and adjectives translated from Russian.

	Literal	Metonymic	Metaphorical
**Nouns: les**	‘Forest’ •*To be covered with forests* •*Siberian forests*	‘Wood’ •*To stock the wood* •*To float the wood downstream*	‘Great number of something raised’ •*Raised bayonets* •*Raised hands*
**perelom**	‘A break or crack in a bone’ •*Vertebral fracture* •*Fracture with displacement*	‘Part of the body with a fracture’ •*To impose a plaster on a fracture* •*The fracture itches*	‘A turning point in something’ •*Historical crisis* •*A turning point in illness*
**more**	‘A large area of salt water, a sea’ •*Baltic sea* •*bottom of the sea*	‘Water in a sea’ •*The sea is warm today* •*To enter the sea knee-deep*	‘A lot of something’ •*A lot of champagne* •*A lot of affairs*
**Verbs:kipet’**	‘A liquid boils’ •*Water boils at 100*° •*C* •*The soup is already boiling*	‘The liquid in the container boils’ •*The kettle is boiling* •*The pot was boiling over the fire*	‘A person feels something such as anger very strongly’ •*He boils with resentment* •*To boil with indignation*
**letet’**	‘To move through the air using wings or special device’ •*A bird is flying* •*A parachute is flying*	‘To travel by plane’ •*We flew for 3 h* •*You are flying over the ocean*	‘To move fast’ •*Where are you rushing?* •*A hare is running through the field*
**krasnet’**	‘To turn red’ •*Berries turn red in August* •*Aspen leaves turn red in autumn*	‘To become visible (about red objects)’ •*Red poppies bloomed in the grass* •*In a bush we saw red berries*	‘To turn red in the face, to blush’ •*He lies and does not blush* •*I blushed for what you have done*
**Adjectives:derev’annyj**	‘Made of wood’ •*Wooden bridge* •*Wooden house*	‘Related to the wood’ •*Wooden architectonics* •*Wooden architecture*	‘Without emotions or stiff’ •*Unemotional voice* •*Uneasy walk*
**hitryj**	‘Cunning (about a person)’ •*Cunning man* •*Cunning trickster*	‘Cunning (about behavior or any manifestation)’ •*Cunning eyes* •*Cunning grin*	‘Intricate’ •*To start an intricate game* •*Intricate lock*
**zelenyj**	‘Green (about a color)’ •*Green paint* •*Green leaves*	‘With lots of plants’ •*Green meadow* •*Green lane*	‘Not experienced, especially because of being young’ •*Green youth* •*Green teenager*

All stimuli are available online^8^.

#### Procedure

**FIGURE 1:**
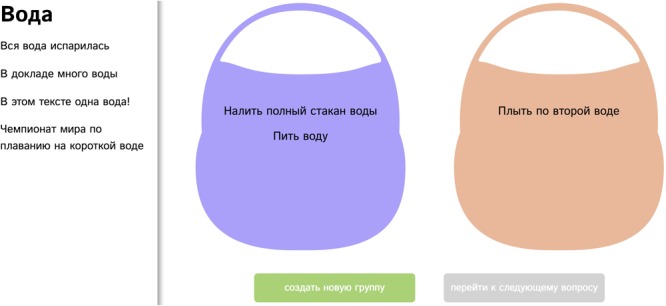
An example trial with the word voda (‘water’). In the left column all phrases available for sorting are listed. In the purple basket there are the phrases *to pour out a full glass of water* and *to drink water*. In the orange basket there is the phrase *to swim in the second pool lane*. Below are the two buttons ‘Create a new group’ and ‘Switch to the next trial’. Participants were not allowed to switch to the next trial until they finished sorting all the phrases from the left column.

An original ‘basket paradigm’ was programmed in Python and Javascript as an online questionnaire^9,10^. Participants were asked to sort several short phrases containing the same word: phrases with the same perceived word sense should be sorted into the same virtual basket (see an example trial with the word «water» in **Figure 1**). The number of baskets (senses) for each word was not pre-defined, participants could create as many baskets as there were phrases in the list, and group the phrases in any way, including placing all the phrases into one basket or placing each phrase into its own basket. Trials corresponding to different polysemous words were presented in random order, but the order of the phrases within the trial was pseudo-randomized for presentation, such that close senses would not be clustered together.

Filling out the full questionnaire took approximately 30 min; participants could interrupt the process at any time and start again where they left off using a personalized link.

#### Analysis

The resulting classifications were analyzed in R (R Core Team, 2016). For linear models, the ‘lme4’ library was used (Bates et al., 2015). For creating tables with linear models’ outcomes (**Tables 3, 5**), we used package sjPlot (Lüdecke, 2017).

The first analysis compared the classifications of participants to the reference classification provided by the lexicographers, and checked whether the degree of similarity was influenced by the participant’s background and by the word class. In this analysis, we built a matrix indexing which senses of the word were clustered together by the participant (see **Table 2**) and then computed the Euclidian distance between the lower triangular part of this matrix and the reference classification matrix provided by the expert lexicographers. A perfect overlap between the matrices corresponded to the Euclidian distance of 0; in our dataset the values ranged from 0 to 7.75, with a mean of 1.74. The resulting set of distances was used as the dependent variable in the linear mixed-effects model; the independent variables included the type of participant (linguists coded as 1, non-linguists as -1), the word class (noun/verb/adjective, treatment contrast coded with noun being the reference level), and the interaction between those factors. The model included random intercepts for participants and words as well as a by-word random slopes for participant type.

**Table 2:** An example classification matrix.

	Phrase 1	Phrase 2	Phrase 3	Phrase 4	Phrase 5	Phrase 6
Phrase 1	1					
Phrase 2	0	1				
Phrase 3	0	1	1			
Phrase 4	0	1	1	1		
Phrase 5	1	0	0	0	1	
Phrase 6	0	1	1	1	0	1

The second analysis aimed to classify the quality of mistakes (defined as a divergence from the lexicographers’ classification of senses) made by the participants. From each person’s classification matrix for each word, we extracted pairs of phrases that the participants grouped together (i.e., all the intersections marked as 1 in **Table 2**). Each phrase in the pairs was labeled as representing literal/metonymic/metaphorical senses, resulting in all possible combinations of the senses. There were three main types of misgroupings: placing literal and metonymic, literal and metaphorical, and metonymic and metaphorical senses together.^11^ We fit three linear mixed-effects models with a binomial link function, with the main effects of word class and participant background as predictors. Each model included random intercepts for participants and words. The dependent variables were: (i) whether literal and metonymic senses of the same word were grouped together; (ii) whether literal and metaphorical senses were grouped together; and (iii) whether metonymic and metaphorical senses were grouped together.

### Semantic Vector Analysis

In addition to the experiment targeting the semantic relatedness of literal/metonymic/metaphorical senses, we performed a semantic similarity estimation of the experimental stimuli using the semantic vectors approach. Semantic vectors for words are obtained from large corpora and capture word co-occurrence statistics in low-dimensional dense vectors; vectors of words that usually occur in the same contexts are close to each other. This closeness, or semantic similarity, has different degrees and can be expressed numerically by a number between 0 (never occurred in the same context and are dissimilar) and 1 (share the same contexts and are identical). The degree of similarity is commonly measured by the cosine similarity measure. This measure is known to be highly correlated with human similarity judgments, showing 0.76 Spearman correlation for Russian (Panchenko et al., 2015) and 0.7 – 0.8 Spearman correlation for English (Baroni et al., 2014).

Semantic vectors were obtained using the word2vec skip-gram algorithm (Mikolov et al., 2013a). This algorithm trains a distributional semantic model on the task of predicting words in context (disregarding their order). Compared to other approaches for building distributional semantic models, such as applying Singular Value Decomposition or Non-negative Matrix Factorization to co-occurrence counts, context-predicting models like word2vec achieve better performance on a wide range of lexical semantics tasks (Baroni et al., 2014).

For this study, word vectors were built from a 2 billion token corpus combining the ruWac Internet corpus (Sharoff, 2006), the Russian online library lib.ru, and the Russian Wikipedia. All words were lower cased and lemmatized, and no stop-word removal was performed. The word2vec Skip-gram model was trained with vector dimension 1024, context window 5 and negative sampling. A very similar model achieved the best results in a semantic similarity evaluation for Russian (Lopukhin et al., 2015; Panchenko et al., 2015). Since we wanted to estimate the semantic similarity between short phrases, we took the average of word vectors in the phrase and built context vectors (Iacobacci et al., 2016). For example, given the phrase ‘wooden bridge,’ vectors for words ‘wooden’ and ‘bridge’ would be averaged to form a single context vector of the same dimensionality. This vector captures the semantic properties of both words in the phrase.

A semantic similarity estimation of the experimental stimuli was performed in the following way: we took our experimental stimuli with literal, metonymic, and metaphorical senses and measured the semantic similarities between all pairs of phrases of the three types. The semantic similarity between phrases was calculated as follows: semantic vectors for all words in the phrase were averaged to form a context vector, and then the cosine similarity was calculated between context vectors. For example, given the two phrases ‘wooden bridge’ and ‘wooden architecture,’ we obtained context vectors for each of these phrases, and then calculated the cosine similarity (a scalar value) between the two context vectors. Then for each word of a particular word class we took all pairs of phrases with any two types of senses (e.g., for the adjective ‘wooden,’ all phrases with a literal sense and all phrases with a metonymic sense) and calculated the cosine similarity between each pair. After that we took the global average. We obtained an average semantic similarity between the two types of senses for a particular word class (in this example, the average semantic similarity between literal and metonymic senses for adjectives). In this way, we obtained the semantic similarity between literal and metonymic, literal and metaphorical, metonymic and metaphorical senses for three word classes: nouns, verbs, and adjectives. Tukey’s HSD test was used to compare the difference between the word classes and between literal-metonymic, literal-metaphorical and metonymic-metaphorical similarity measures.

## Results

### Experimental Results

**FIGURE 2:**
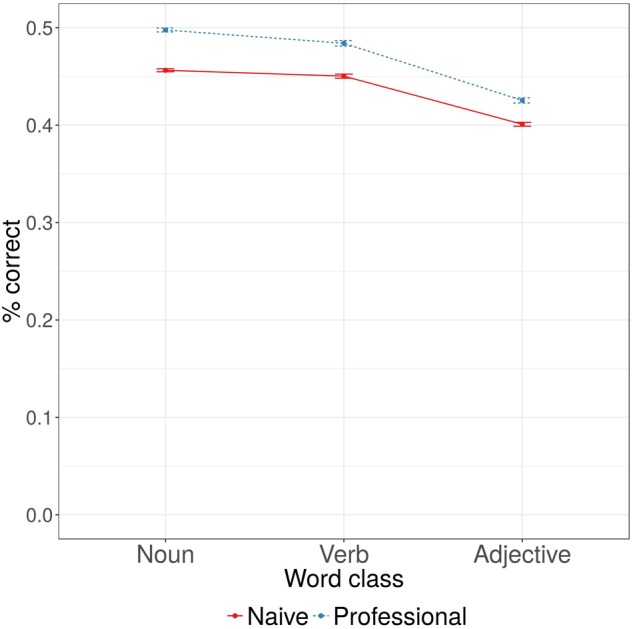
Proportions of correct classifications (compared to the reference classification provided by the expert lexicographers), listed by participant type and word class.

The first analysis of experimental data demonstrated that professional linguists had lower Euclidian distance to the reference classification matrix (see **Table 3** for statistical comparisons), i.e., classified words’ senses more like experts (see **Figure 2**). There was no effect of word class: verbs and adjectives did not have greater Euclidian distance to the reference classification matrix than nouns. We found an interaction between the effects of word class and participant type: naive participants performed closer to the professional linguists in adjective classifications. No other interactions were significant.

**Table 3:** The influence of word class, participant type, and their interactions on Euclidean distance scores.

	Euclidean score		
	Estimate	Standard error	*p*
**Fixed parts**			
(Intercept)	1.613	0.125	<0.001
Adjective	0.357	0.216	0.098
Verb	0.016	0.216	0.942
Participant type	–0.099	0.015	<0.001
Adjective × Type	0.032	0.015	0.030
Verb × Type	–0.004	0.015	0.796
**Random parts**			
σ^2^		0.617	
τ_00,person.id_		0.251	
τ_00,set_		0.001	
*N*_person.id_		2080	
*N*_set_		48	
Observations		57036	
*R*^2^/Ω_0_^2^		0.528/0.528	

Proportions of sense categorizations by sense types are presented in **Table 4**.

**Table 4:** Proportions of classification groupings out of all classifications made by participants, split by word class.

	Word class		
Grouping	Noun	Verb	Adjective
Literal+literal	0.13	0.15	0.12
Literal+metaphor	0.03	0.01	0.03
Literal+metonymy	0.36	0.39	0.22
Metaphor+metaphor	0.16	0.16	0.15
Metaphor+metonymy	0.07	0.06	0.25
Metonymy+metonymy	0.18	0.15	0.14
Other (grouping two different literal senses, or two different metonymies of the same literal sense, etc.)	0.07	0.08	0.09

*Correct groupings are shaded in gray.*

**FIGURE 3:**
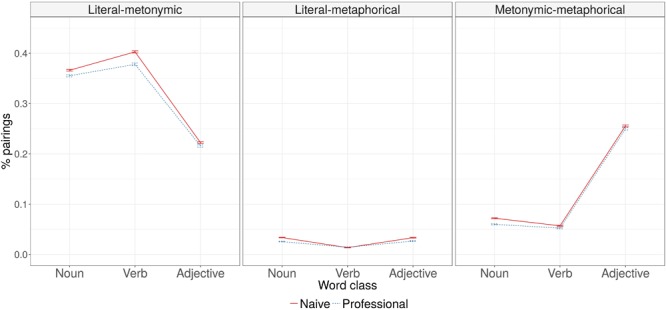
Proportions of misclassifications by participant type and word class.

Analysis of misclassifications made in the experiment (see **Figure 3**) showed that the probability of literal and metonymic senses of the word being grouped together was lower in adjectives than in nouns (for all statistical comparisons, see **Table 5**). Naive participants were more likely to perform these types of misclassifications. There was a significant interaction between participant type and word class: naive participants were more likely to misclassify verbs.

**Table 5:** The influence of word class, participant type, and their interactions on the probability of three types of misclassifications.

	Literal-metonymic			Literal-metaphorical			Metonymic-metaphorical		
	Estimate	Standard error	*p*	Estimate	Standard error	*p*	Estimate	Standard error	*p*
**Fixed parts**					
(Intercept)	–0.989	0.151	<0.001	–4.843	0.226	<0.001	–3.601	0.195	<0.001
Adjective	–1.004	0.193	<0.001	–0.359	0.376	0.339	1.715	0.257	<0.001
Verb	0.362	0.222	0.103	–0.734	0.405	0.070	–0.695	0.271	0.010
Participant type	–0.033	0.010	0.002	–0.235	0.038	<0.001	–0.138	0.017	<0.001
Adjective × Type	–0.003	0.007	0.671	0.072	0.019	<0.001	0.096	0.010	<0.001
Verb × Type	–0.045	0.007	<0.001	0.151	0.025	<0.001	0.078	0.015	<0.001
**Random parts**					
τ_00,person.id_		0.150			1.866			0.362	
τ_00,set_		1.713			1.565			2.130	
*N*_person.id_		2080			2080			2080	
*N*_set_		48			48			48	
Observations		706742			706742			706742	
Deviance		785622.444			128866.874			362347.794	

The probability of literal and metaphorical senses of the word being grouped together was higher in naive participants. In addition, there was an interaction between participant type and word class: naive participants made fewer mistakes in verb and adjective classification than in classification of the other word classes.

The model accounting for grouping metonymic and metaphorical word senses revealed that naive participants were more likely to create misgroupings. We also found a main effect of word class: adjectives were more likely than nouns to be misclassified, while verbs on the contrary, were less likely. In addition, an interaction was found between the main effects of participant type and word class: naive participants were less likely to misclassify adjectives and verbs than nouns.^12^

In sum, we found that both professional and naive participants preferred to group together literal and metonymic senses of nouns, verbs, and adjectives. Moreover, metonymic and metaphorical senses of adjectives, but not those of nouns or verbs, were also often grouped together. In contrast, literal and metaphorical senses were rarely classified together. These results provide evidence that literal and metonymic senses are perceived to be related in all the three word classes. The experiment also revealed that in adjectives non-literal (metonymic and metaphorical) senses were perceived to be related more than in nouns or verbs. We also confirmed that participant background influences accuracy: professional linguists grouped the stimuli more like expert lexicographers, while non-linguists were consistently less accurate in all three word classes. Non-linguists misgrouped all types of senses more often than linguists. However, no evidence was found in favor of different classification strategies used by the naive participants and the professional linguists; both groups showed very similar classification and misclassification patterns.

### Semantic Similarity Evaluation

**FIGURE 4:**
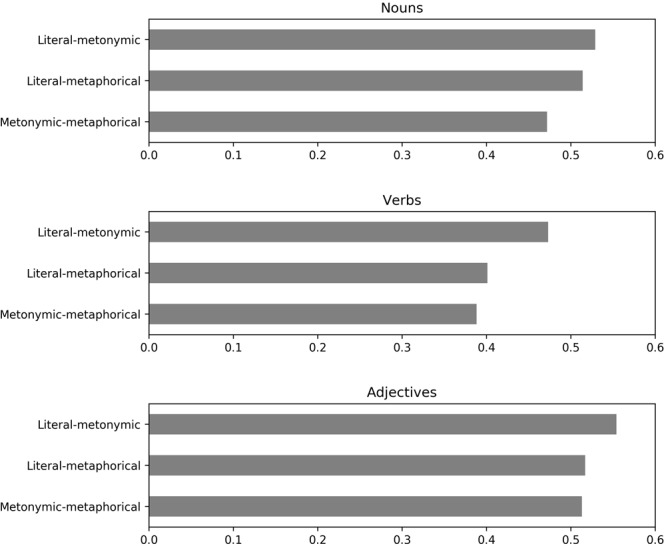
Cosine similarity for literal, metonymic, and metaphorical senses in nouns, verbs, and adjectives.

The semantic vector analysis produced corpora-based values of cosine similarity between pairs of literal, metonymic, and metaphorical senses of the same 48 polysemous words that were used in the experiment (see **Figure 4**). The analysis showed that, overall, the semantic similarity between all types of senses was higher in adjectives than in nouns and verbs (adjectives-nouns, *p* = 0.04; adjectives-verbs, *p* = 0.001). Verbs had the lowest degree of similarity among all the word classes (verbs-nouns, *p* = 0.001; verbs-adjectives, *p* = 0.001). Critically, we found that for all three word classes the similarity between literal and metonymic senses was significantly higher than the similarity between the other types of senses (reaching the greatest cosine distance values of 0.53 in nouns, 0.47 in verbs and 0.55 in adjectives).^13^ For nouns, the literal-metonymic similarity was significantly higher than the metonymic-metaphorical similarity (*p* = 0.001). The same was true for verbs (*p* = 0.001) and adjectives (*p* = 0.02). Moreover, for verbs but not for nouns and adjectives the literal-metonymic similarity was higher than the literal-metaphorical similarity (in verbs *p* = 0.004). For nouns but not for verbs and adjectives, the literal-metaphorical similarity was higher than the metonymic-metaphorical similarity (*p* = 0.0015).^14^

The results of the semantic similarity analysis are in line with what was found in the experiment: phrases with literal and metonymic senses, which the participants preferred to group together, had the highest degree of cosine similarity (i.e., they share more contexts in the corpora). Moreover, the metonymic and metaphorical senses of adjectives, which were grouped together in the experiment significantly more frequently than the metonymic and metaphorical senses of nouns and verbs (25% of cases, see **Table 4**), had the highest degree of similarity among all word classes (0.51 in adjectives vs. 0.47 in nouns and 0.39 in verbs).

## Discussion

The goal of the present study was to test whether different senses of polysemous words are stored in the mental lexicon separately or if their representations overlap, and whether it depends on a word’s class. We ran a semantic clustering experiment in which participants with linguistic and non-linguistic backgrounds were asked to sort short phrases with literal, metonymic, and metaphorical senses of a word. The exploited sorting task was sensitive to semantics: the unlimited time people could spend comparing stimuli and the unrestricted number of groups made it possible to capture semantic overlap and semantic differences between the senses. The task provided a robust means to examine which types of word senses are more likely to be grouped together. The key finding was that literal and metonymic senses of the three examined word classes (nouns, verbs, and adjectives) were grouped together more often than other senses. In addition, we found that in adjectives, metonymic and metaphorical senses were sorted together more often than in the other word classes. The first finding is consistent with the suggestion of Klepousniotou and Baum (2007) and Klepousniotou et al. (2008) that literal senses overlap more with metonymic than with metaphorical senses. The second finding suggests that the results obtained for one word class cannot be generalized on another: the results for adjectives differed from the results for nouns and verbs. We also measured the distributional semantic similarity between the stimuli with different types of senses. In the following discussion, we first compare the experimental and the semantic similarity estimation results; we then propose a view on how polysemous words might be stored in the mental lexicon, discuss the difference in the results for nouns and verbs vs. adjectives, and compare our findings to the results of the categorization task by Klein and Murphy (2002). Moreover, we discuss the performance of the participants with and without a linguistic background.

Although Klein and Murphy (2001) proposed a distinction between semantic relatedness and semantic similarity, arguing that historically related senses may be conceptually very different (as in the literal and metonymic senses in the example *His paper was boring* ‘content written on the paper’/*The paper fired half its reporters* ‘the publisher of a newspaper’), our results do not support this view. We compared the results of the sorting task with the results of the semantic vector analysis and found that those types of senses that the participants preferred to group together (which we interpret as semantic relatedness) also has the highest degree of similarity, sharing more contexts in the corpora. Specifically, phrases with literal and metonymic senses were often sorted in the same virtual basket and proved to be more similar than other types of senses based on the results of the vector analysis. Moreover, we found that the participants often classified together phrases with adjectives that have metonymic and metaphorical senses. The distributional analysis mirrors the experimental results: for adjectives, the semantic similarity between metonymic and metaphorical senses was higher than it was for both nouns and verbs. Although our corpora analysis focused on the distributional similarity of the stimuli, while Klein and Murphy (2001) discussed the conceptual similarity, we think that the conceptual similarity implies the distributional similarity. Words that are conceptually similar (synonyms, co-hyponyms, etc.), are interchangeable in context and thus are also distributionally similar. Our study shows that the notions of semantic relatedness and semantic similarity are very close. It suggests that in our experiment the participants were probably guided not only by the perceived relatedness of senses but also by their similarity. We doubt that the two measures should be contrasted. Furthermore, we suggest that two senses of a word are perceived as close only when they share many contexts: it is distributional similarity that underlies semantic overlap. Several experiments have shown that people can learn the meaning of a new word based on contexts with this word (McDonald and Ramscar, 2001; Erk, 2016; Kuperman and Snefjella, 2017). New senses of polysemous words might also be learned from context. If the contexts are similar to those of the already known sense, the new sense will be treated as being close to the already known sense and will be stored in the same representation; if not, the new sense will have a separate representation in the mental lexicon. If this argument is correct, then sense relatedness comes from contextual similarity and not vice versa.

In the section “Introduction,” we gave an overview of different accounts of multiple sense storage in the mental lexicon. As our offline experiment was focused on the participants’ sorting decisions and did not force them to speed up — participants could spend as much time as they needed considering each stimulus — we believe that it captures the way polysemous words are represented in the mental lexicon. Previous studies have shown that online and offline experiments with the same set of stimuli provide comparable results and that offline tasks are more sensitive to semantics (see Klein and Murphy, 2002). Our results may shed light on the organization of the senses of polysemous words in the mental lexicon. One possibility, that words have a list of senses that are stored separately (the separate sense account), should have led to a separate grouping of contexts with different senses. However, that is not what we observed in the experiment: participants grouped literal and metonymic senses together in 36% of cases for nouns, 39% for verbs and 22% for adjectives. For nouns and verbs, the groups of ‘literal + metonymic’ senses even got the highest proportion of all the misgroupings. Another possibility is that all of the senses are stored in one core representation and are derived via semantic rules (the single sense account). In that case we should have observed various misgroupings of literal, metonymic, and metaphorical senses. But our data show that literal and metaphorical senses are rarely grouped together: only 3% of cases for nouns, 1% for verbs and 3% for adjectives. As for metaphorical and metonymic misgroupings, they are rare for nouns and verbs (7% of cases for nouns and 6% for verbs) but relatively frequent — 25% of cases — for adjectives. A final possibility is the hybrid approach to sense storage: some senses may be close, stored in one representation and derived on demand while others may have separate representations. The hybrid approach should lead to the following pattern: close senses from one mental representation should be consistently grouped together while distinct senses should be sorted in separate groups. Our results for nouns and verbs follow this pattern and thus support the hybrid account. Polysemy seems to be a continuum in which literal and metonymic senses are closer to each other and may be stored together while metaphorical senses are less similar and are stored separately.

However, for adjectives the classification pattern was found to be different: literal senses were often grouped together with metonymic ones, and metonymic senses were grouped with metaphorical ones. Adjectives seem to have a high overlap of all three types of senses. It can also be indirectly supported by the fact that, overall, participants struggled more with adjective classification than with the other word classes (mean classification accuracy for adjectives was 41%, vs. 47% for nouns and 46% for verbs). The more similar the senses are, the harder it was to distinguish them. This result is inconsistent with any existing account of multiple sense storage. The difference between adjectives on the one hand and nouns and verbs on the other may be explained by the fact that adjectives are much less concrete, imageable or frequent than nouns or verbs (see Babaeva, 2010). Nouns usually refer to real world objects (like *okno* ‘window,’ *kniga* ‘book’ or *lisa* ‘fox’ in our experiment) and have a set of semantic features that allow us to imagine the referent. In the case of metonymic extensions, the focus of attention shifts from one aspect of an object to another, like in animal/product derivation for *lisa* ‘fox.’ In metaphorical derivation the bearer of the property changes; for the word *lisa* ‘fox,’ the property ‘being cunning and sly’ is carried from an animal to a person. Most of the verbs denote actions or states and have a number of arguments that also allow us to imagine the situation referred to with a verb (like *kipet’* ‘to boil’ or *resat’* ‘to cut’). Moreover, in verbs regular metonymic or metaphorical extensions are usually accompanied by a change in the argument structure, like *voda kipit* ‘the water boils’/*on kipit ot zlosti* ‘he boils with rage’ (metaphorical shift) or *ona rezhet pirog nozhom* ‘she cuts the pie with a knife’/*etot nozh horosho rezhet* ‘the knife cuts well’ (metonymic shift). These changes signal a shift in meaning. As for adjectives, they provide information about some qualities of a noun (like *zelenyj* ‘green’ or *grubyj* ‘rough’) and are hard or even impossible to imagine separately from an object. Language speakers probably are not used to considering adjectives as something separate from nouns, and cannot estimate their senses separately, which might lead to adjectives having semantic representations different from those of nouns or verbs. The difference between word classes that we found in our experiment implies that the conclusions about sense representation in the mental lexicon generated in previous studies with nouns and verbs cannot be automatically extended to polysemy of other word classes. We also think that it is important to broaden future experimental studies of polysemy by including other word classes.

The findings of our experiment are in stark contrast to the results of Klein and Murphy (2002). In the categorization task with two alternatives, Klein and Murphy (2002) found that participants preferred not to categorize together different senses of a word, even after being primed with a polysemous relation. Instead, participants chose either taxonomic or thematic alternatives. Klein and Murphy (2002) concluded that it was consistent with the view that senses of a polysemous word had little semantic overlap and should be stored separately in the mental lexicon. However, the polysemous stimuli used by Klein and Murphy were not divided into metonymic and metaphorical categories. As Klepousniotou et al. (2008) noticed, in the original experiment some items were metonymically polysemous (i.e., *book*) while at least half were metaphorically polysemous (i.e., *atmosphere*). Klepousniotou and colleagues explained the results of the experiments by Klein and Murphy (2001, 2002) by a mixture of word types in the stimulus set. Indeed, our experiment showed that participants preferred not to mix the metaphorical sense with either literal or metonymic senses, except in the case of adjectives. The mixture of different types of polysemy may have caused inconsistency in the responses of the participants in Klein and Murphy (2002) and the 20% of cases when the two different senses of a word were categorized together may be explained by metonymies in the dataset.

Moreover, we believe that in addition to the separate analysis of literal, metonymic, and metaphorical senses, it might be interesting to focus on a systematic comparison of regular polysemy patterns (e.g., a container/containee metonymic pattern or an animal/person metaphorical pattern). The strength of the relationship between two senses may influence participants’ decisions more than the polysemy type. Jager and Cleland (2015) found that in the lexical decision task metonymies were recognized slower than metaphors and they assumed that nouns with an animal/product metonymic extension that were used in their experiment had developed separate (competing) entries in the mental lexicon. This could happen because the relationship between animals and the products derived from them may have been lost due to urban lifestyles. On the contrary, the link between animal/person metaphorical extensions (like *donkey* or *tortoise*) may strengthen over time. Jager and Cleland concluded that the degree of relationship regularity (i.e., metonymy = regular sense relationship, metaphor = irregular sense relationship) and semantic overlap are not inherent in specific semantic extensions. Both metonymic and metaphorical senses could be derived by means of regular or irregular rules and both could have small or large degrees of semantic overlap. We suggest that in the future it might be fruitful to pay more attention to regular polysemy patterns in order to discover whether polysemous words that exhibit different polysemy patterns are indeed stored differently in the mental lexicon.

Another important issue that the previous studies have not addressed is the choice of participants. In most (or even all) of the experiments, the participants were European and American university students. Henrich et al. (2010) showed that Western, Educated, Industrialized, Rich, and Democratic (WEIRD) societies are the least representative populations one could find for generalizing about humans. However, even within these populations people with linguistic and non-linguistic educational backgrounds may give different answers in linguistic experiments. In the current study we compared how people with and without a linguistic background clustered senses. We expected that the former would think about senses of a word in terms of ‘metonymy’ and ‘metaphor’ and thus their classifications would be close to the reference classification provided by the lexicographers, while the latter would sort senses according to their perceived relatedness that may differ from the reference classification. The results showed that participants with a linguistic background did respond more like experts: their mean classification accuracy was higher as compared to that of participants with a non-linguistic background. Even in the group of professionals, however, the mean classification accuracy varied from 0.43 in verbs to 0.50 in nouns, which is not very close to the expert classification. The low accuracy can probably be explained by our relatively vague classification criteria: not only linguists but also journalists, translators, and interpreters were included in the group of participants with a linguistic background. These individuals might not think of word senses in terms of metonymy and metaphor and might therefore perform closer to naive participants.

Another important conclusion from our study is that although naive participants struggled more than professionals with all types of senses, we did not find any pattern that would distinguish people with a linguistic background from people without a linguistic background. Similar classification patterns imply that the perceived relatedness of senses that we suppose to be at the heart of the naive classification is close to the metonymic and metaphorical distinctions that professionals probably rely on. These results have two implications. One is that sense relatedness seems to correlate with the type of polysemy: literal and metonymic senses are perceived as close, while literal and metaphorical senses are not. The second implication is that we should be careful when selecting participants for linguistic experiments. People with a linguistic background seem to use their linguistic knowledge, and their answers are more similar to what experimenters expect, which may not allow experimenters to generalize about all native speakers.

## Conclusion

We have demonstrated that literal and metonymic senses of polysemous nouns and verbs are semantically related, share many similar contexts, and thus might be stored in the unified lexical representation in the mental lexicon. In contrast, metaphorical senses are perceived as unrelated, share fewer contexts, and are more likely to have distinct representations. We also found that the conclusions concerning nouns and verbs do not extend to the polysemy of adjectives. In adjectives, metonymic senses significantly overlap with literal senses on the one hand and metaphorical senses on the other, which was not found in either nouns or verbs. Further work might shed light on the form of the sense representation of adjectives in the mental lexicon. In addition, we showed that people with linguistic backgrounds perform more like experts than people without linguistic backgrounds, but both groups still share principal patterns of sense classification. Experimenters should take this observation into account when selecting participants for their studies if they intend to generalize their results to the whole population.

## Ethics Statement

All participants gave informed consent in accordance with the Declaration of Helsinki. We did not seek approval by an institutional review board for the experiment because it is not required for a study of the type reported in this manuscript.

## Author Contributions

Conception and design of the semantic clustering experiment: ALa and OD. Programming of the experiment: KL and ALa. Data collection: ALa and ALo. Statistical analysis of experimental results: ALa. Semantic vector analysis: KL. Preparation of the final document: ALo, ALa, KL, and OD.

## Conflict of Interest Statement

KL was employed by the company Scrapinghub. The other authors declare that the research was conducted in the absence of any commercial or financial relationships that could be construed as a potential conflict of interest.

## Footnotes

1 We will follow linguistic terminology and call different meanings of polysemous words senses, while generally referring to homonyms as having different meanings. However, meaning will also be used as a term covering both cases.

2 The mental dictionary contains comprehensive linguistic information — phonological, morphological, semantic, and syntactic — that speakers know about individual words and/or morphemes (Emmorey and Fromkin, 1988; Aitchison, 2012).

3 In homonymy a lexical item accidentally carries two (or more) distinct and unrelated meanings, such as *bank 1* which means ‘a financial institution’ and *bank 2* which means ‘the side of the river’ (Lyons, 1977).

4 http://www.ldoceonline.com/

5 In this study, participants were not selected or invited to the study based on their gender. The fact that less than 0.5% of participants were male can have two explanations: first, women are more likely than men to volunteer (Freeman, 1997), and second, among participants with linguistic background there are more women than men.

6 This classification contains at least one simplification: students of linguistics and related fields who had not yet obtained their degree were considered to be naive.

7 http://www.ruscorpora.ru/en/

8 https://osf.io/x59ke/

9 http://sgs.ll-cl.org/

10 https://github.com/lopuhin/sense-grouping-survey

11 There was a small number of other misclassifications, namely classifying two different literal senses or two different metonymic senses together, but we did not include these in our analysis.

12 When preparing this paper for publication, we discovered a possible confound in the stimuli: in eight words (two nouns, two verbs, and four adjectives) metonymic senses were derived from metaphorical senses while in the other 40 words metonymic senses were derived from literal senses. We excluded these eight words from the analyses and reanalyzed the participants’ correct classifications and misclassifications: https://osf.io/63fn9/. All but two effects are the same: in the correct classifications analysis, we did not find the interaction between the effects of word class and participant type within adjectives; in the misclassification analysis, verbs did not differ from nouns in the amount of metonymic-metaphorical misclassifications. These changes do not influence our conclusions.

13 For reference, the cosine similarity between frequent synonyms is between 0.5 and 0.95.

14 All data and analyses are available online: https://osf.io/staew/.
